# Granulometric Analysis After Spontaneous Disintegration of Tablets Containing Micronized Purified Flavonoid Fraction (SIZE Study)

**DOI:** 10.7759/cureus.100169

**Published:** 2025-12-27

**Authors:** João Vitor Santana da Silva, Adriane dos Santos Francisco, Isabella Mourão Machado Valle, Juliana Alves Ferreira, Raianne Rebelo, Estefane Theophilo de Freitas Pereira, Renata Lima, Luiz Cláudio Rodrigues Pereira da Silva

**Affiliations:** 1 Faculdade de Farmácia, Universidade Federal do Rio de Janeiro, Rio de Janeiro, BRA; 2 Medical Affairs, Servier Brazil, Rio de Janeiro, BRA

**Keywords:** absorption, desintegration, diosmin, micronized purified fraction flavonoid, particle size

## Abstract

Background: Micronized purified flavonoid fraction (MPFF), primarily composed of diosmin and hesperidin, is used to treat chronic venous insufficiency by improving venous function. Traditional diosmin suffers from low bioavailability due to poor solubility, but micronization enhances absorption by reducing particle size. This advancement boosts therapeutic effectiveness, crucial for relieving venous disorder symptoms. Nevertheless, inconsistencies in micronization across manufacturers can affect drug effectiveness, highlighting the need for standardized processes.

Objectives: This study, called the SIZE study, aims to evaluate the particle size distribution of MPFF tablets containing diosmin/hesperidin (900/100 mg) from four Brazilian pharmaceutical manufacturers.

Methods: The spontaneous disintegration method was used to simulate the dissolution process, and granulometric analysis was performed using optical microscopy and image analysis. Samples were collected at 5 and 15 minutes post-disintegration, and particle sizes were categorized into ranges. The spontaneous disintegration method used was not based on the pharmacopoeia, but rather on a study published in 2018.

Results: Statistically significant differences were found between the products, particularly in the 1-5 µm range, with the reference product (Product I) showing the highest percentage of fine particles. This finer granulometry likely contributes to faster dissolution and improved absorption.

Conclusions: The SIZE study highlights the importance of particle size in drug solubility and bioavailability, suggesting that differences in the micronization process affect therapeutic efficacy. The findings underscore the need for standardized regulations on micronization to ensure product consistency.

## Introduction

Micronized purified flavonoid fraction (MPFF), which is composed of 90% diosmin and 10% hesperidin, has been widely used in the management of chronic venous disease (CVD), and its efficacy is largely attributed to its ability to improve venous tone, reduce venous capacitance, and decrease capillary hyperpermeability, along with its anti-inflammatory action [1-4]. These effects are crucial for alleviating symptoms like leg pain, edema, and a sensation of heaviness, which are common in patients with venous disorders [5].

Diosmin is a naturally occurring flavonoid, derived primarily from citrus fruits, which has been extensively studied for its potential benefits in treating chronic venous disease (CVD), hemorrhoidal disease, and other vascular disorders. One of the fundamental aspects of diosmin is related to its low absorption in the gastrointestinal tract due to its low water solubility [6]. Low bioavailability leads to reduced efficacy, necessitating higher doses to achieve therapeutic effects, which can increase the risk of side effects. To address these limitations, diosmin is processed through micronization, a technological approach aimed at reducing particle size to an average diameter of around 2 µm. This process significantly enhances the dissolution rate of the active ingredient, thereby increasing its absorption and metabolism, ultimately leading to improved therapeutic effectiveness [1,6-7].

The importance of particle size in drug solubility and bioavailability is well-documented in the pharmaceutical literature. Smaller particle sizes result in a larger surface area available for dissolution, which accelerates the dissolution process and enhances absorption in the gastrointestinal tract [7]. Studies have demonstrated that the micronization of diosmin to achieve particle sizes predominantly below 5 µm results in a marked improvement in therapeutic outcomes, particularly for chronic venous disorders where rapid drug action is essential for symptom relief [8]. Preclinical and clinical pharmacological studies have demonstrated the beneficial impact of micronization on the pharmacological activity of MPFF [9-11].

Zupanets et al. compared the degree of micronization of the MPFF in the reference drug from France and two other medications from Ukraine. Significant differences in particle size were found among various manufacturers, suggesting that there may be differences in the clinical performance of different products due to different post-disintegration granulometry. Despite the promising benefits of micronization, the consistency of the micronization process across different manufacturers remains a concern. Variability in particle size can lead to significant differences in drug dissolution rates and, consequently, therapeutic efficacy [7].

The objective of the present work is to describe a comparative granulometric evaluation of tablets containing 1000 mg of MPFF based on diosmin/hesperidin (900/100 mg) that were subjected to spontaneous disintegration. Four medications from different pharmaceutical manufacturers (named as I, II, III and IV), containing diosmin/hesperidin (900/100 mg), were purchased directly from Brazilian pharmacy stores. One of them was the reference product, Daflon^® ^1000 mg tablets from Laboratórios Servier do Brasil (named as product I). The current work provides a deeper understanding of the granulometric characteristics of these formulations and their potential correlation with therapeutic efficacy.

## Materials and methods

Materials

The reference pioneer medication was named as product I from Servier Laboratories. The other diosmin-hesperidins were named as products II, III and IV, from other Brazilian pharmaceutical manufacturers. For the purpose of this report, other diosmin-hesperidins are those that contain the same drugs and dosages as the product I, but do not have the bioequivalence certification that would classify them as “similar” or “generic”, according to ANVISA (Agência Nacional de Vigilância Sanitária) definitions, and are therefore classified as “specific drugs” in Brazil, since they cannot be tested for bioequivalence against a comparator product [12].

Methods

Spontaneous Disintegration and Sampling

The methodology was based on the spontaneous disintegration protocol published by Zupanets et al. (2018) [7]. The experiment consisted of four cups containing 250 mL of distilled water, each containing one tablet of each drug, with the disintegration time recorded by a stopwatch and samples collected at 5 and 15 minutes, with 2 mL samples collected from three different points in the cup (bottom, central position in the vertical direction, and near the water surface).

This process was performed five times, i.e., each of the four drugs had five tablets in the experiment, according to the protocol described in the previous paragraph. Therefore, the complete study included 120 individual samples and analyses, with four drugs, five tablets each, at two time points (5 and 15 minutes), and three points in each glass (bottom, center, and surface). This design was implemented to improve the statistical consistency of the study.

Visual Evaluation of Particle Diameters by Optical Microscopy and Image Analysis

Four drops of each sample were carefully placed at the center of the glass microscope slides, which were then covered with cover slips and observed under a microscope at 100X magnification. The images were captured by a camera attached to the microscope and stored on an online data storage platform. The images were analyzed using ImageJ software (https://imagej.net/ij/), a well-established free tool for microscopy analysis, for counting and measuring particle diameters [13]. The granulometric analyses involved evaluating the quantity, morphology, and dimensions of particles resulting from the disintegration of tablets containing MPFF. Particle count data were tabulated and organized into five diameter ranges (1 to 5 μm; 5 to 10 μm; 10 to 20 μm; 20 to 50 μm, and above 50 μm) to obtain the population distribution of particles by size ranges. The data were expressed as the mean and standard deviation of the replicates.

The images were pre-processed to remove noise and enhance the characteristics of the particles of interest using adaptive methods to distinguish the particles from the image background. The commands from the Process > Binary > Make Binary menus were used. Then, a scale of 1 μm/pixel was set in the Set Scale menu. This ratio was obtained from a previous calibration of a slide with a micrometer scale. After segmentation, the particles were analyzed using the Analyze > Analyze Particles function of the ImageJ software. This function allows the measurement of various properties of the particles, including area. Assuming that the observed particles predominantly exhibited a circular morphology, the following formula was applied to determine the diameters expressed as:

ø=2√(A/π)

where ø is the circle's diameter, A is the area, and the value of π is 3,14.

Statistical analysis

The statistical analysis of the data was conducted using GraphPad Prism software (https://www.graphpad.com/) with a free trial period, with results expressed as mean ± standard deviation [14]. A value of p ≤ 0.05 was considered statistically significant. Outlier exclusion was performed using Grubbs' Test, considering a significance level of 95%. To investigate significant differences between the medication groups in relation to each established particle size range, statistical analyses were applied. Initially, two-way ANOVA was used to evaluate the influence of different medications and particle size ranges. Then, Tukey's post-hoc test was conducted to identify significant differences between the medication groups.

## Results

**Figure 1:**
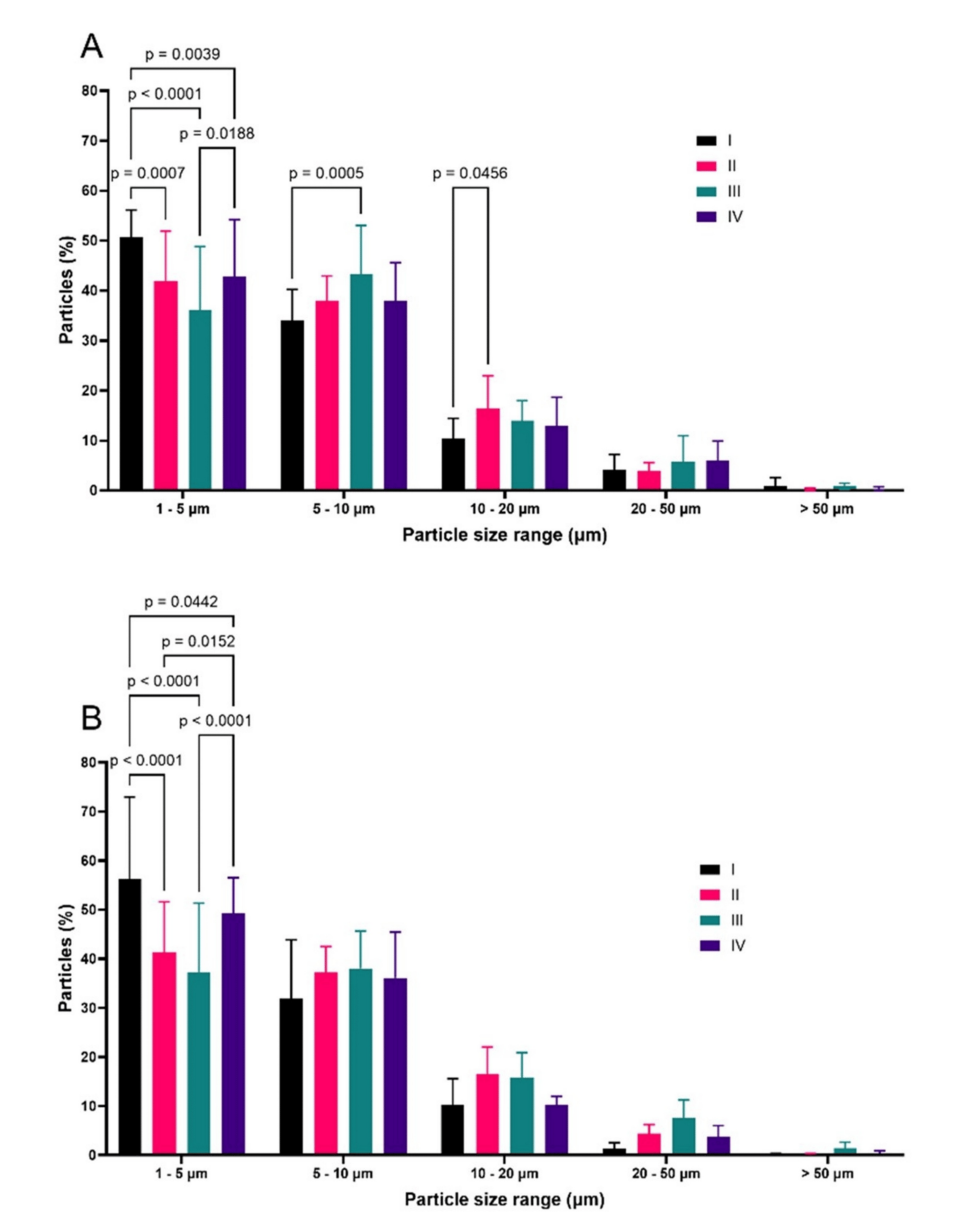
Percentage distribution of particles in relation to size ranges for 1000 mg medications (products I-IV) After 5 minutes (A), and 15 minutes (B) of disintegration (n = 15, for each medication).

The data obtained for all medications revealed that the predominant particle sizes ranged from 1 to 50 µm. To evaluate medication differences more critically, size ranges were defined and grouped as follows: 1-5 µm; 5-10 µm; 10-20 µm; 20-50 µm; and > 50 µm. The results, organized by size range, were normalized by the total number of particles per range, allowing a graphical representation of percentages. The results showed statistically significant differences between medications in terms of particle size distribution across various ranges (Figures 1A, 1B). It is noteworthy that the reference medication (product I) showed a significantly higher percentage of particles in the 1-5 µm range compared to the other medications at both evaluated time points.

At the 5-minute time point (Figure 1A), in the 1 to 5 µm range, the reference product had an average of 50.8 ± 5.3% of particles, higher (p-values < 0.005) than product II (41.8 ± 10.1%), product III (36.1 ± 12.7%), and product IV (42.9 ± 11.3%). For larger particles (Figure 1A), the behavior of the medications reversed, since product I presented the lowest percentage of particles between 10 and 20 µm. The most notable difference was observed in the comparison of products I and II (p < 0.05). This suggests that the reference product may offer better absorption speed and extent compared to product II. This is because one of the aims of the micronization of pharmaceutical ingredients for oral use is to reduce particle size to the point that there is an increase in dissolution rate and consequently in drug bioavailability. Generally, particles between 1 and 10 micrometers are desirable in micronization processes because, compared to larger bulk particles, they have a greater surface area in contact with the dissolution medium and a thinner diffusion layer, explaining the improvement in performance. At the 15-minute mark, the results continued to demonstrate the superiority of product I compared to other diosmin-hesperidin similar medications, as it had the highest percentage (56.3 ± 16.6%) of particles in the 1 to 5 µm range (Figure 1B). The most evident differences were found in comparisons between I vs. II and I vs. III, both with p < 0.0001.

Product II had 41.3 ± 10.3% of particles in the 1 to 5 µm range, and product III had only 37.3 ± 14.1%. Although product IV also showed statistically lower values than product I in this size range (49.3 ± 7.2%), the p-value was 0.0442. At the 5-minute time point, product I also showed the highest particle population percentage in the expected micrometric size range after 15 minutes of disintegration tests. In the other size ranges evaluated, product I did not show a different distribution from the other medications from a statistical point of view. However, it is clear that the reference product has lower percentages of particles in the ranges above 10 µm.

**Figure 2:**
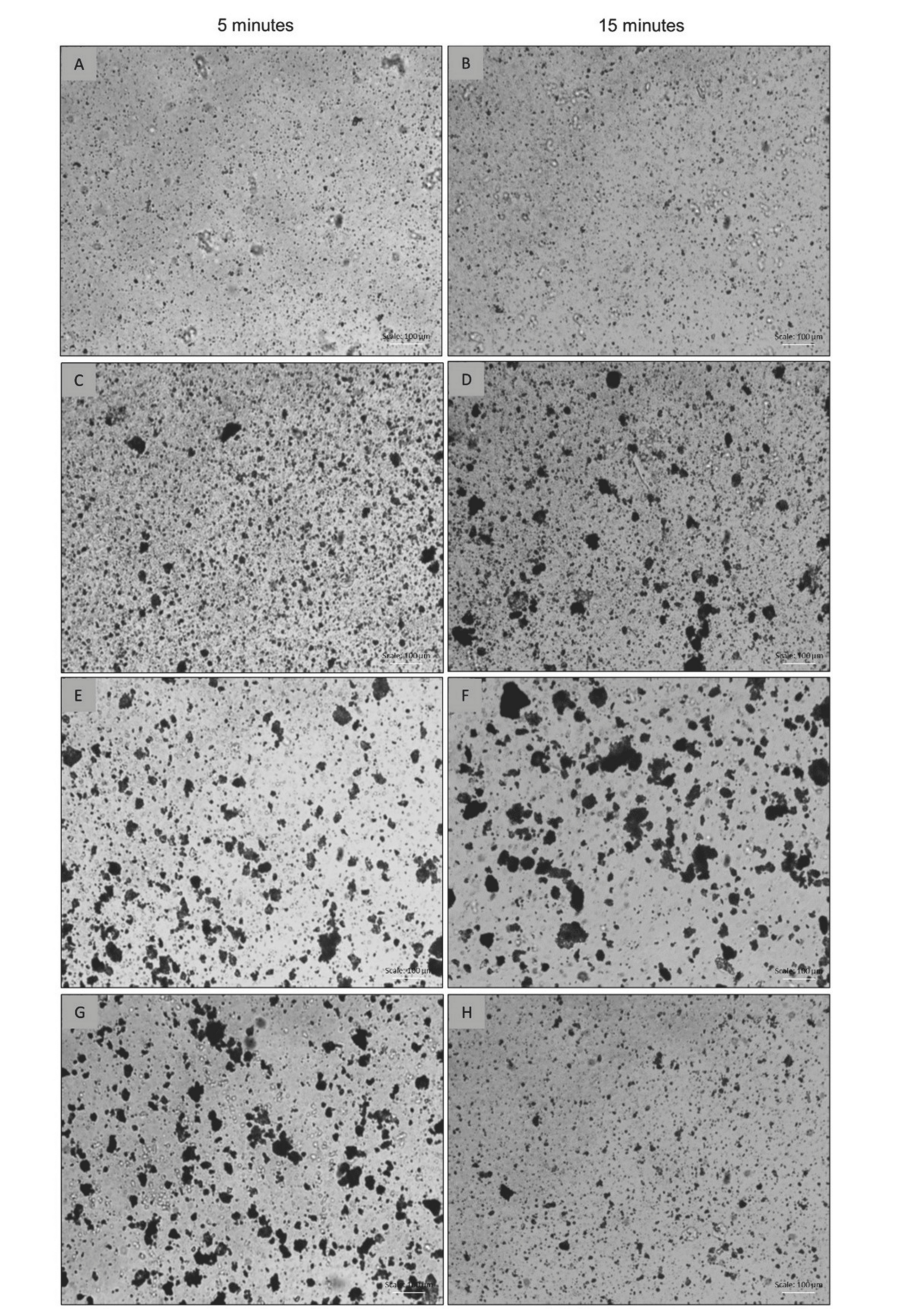
Optical microscopy of particle dispersions from the disintegration of 1000 mg medications Product I (A and B), Product II (C and D), Product III (E and F), Product IV (G and H). Scale bar = 100 µm (bottom right corner).

Microscopy images (Figure 2) unequivocally demonstrate that product I, after 5 minutes of disintegration, generates finely divided particles, especially compared to products II and IV. Product II also exhibited less coarsely divided particles than III and IV, but in comparison to the reference product, it still has larger particles in greater proportion. This variability in particle size distribution may be attributed to differences in micronization technologies employed in the production of the active ingredients. Therefore, these results highlight the importance of precise granulometric characterization in assessing the quality and consistency of micronized pharmaceutical formulations. The 15-minute test showed a peculiarity compared to the 5-minute test, as all medications showed an increase in the number of particles in the smallest size range (1 to 5 µm) (Figures 2B, 2D, 2F, 2H). Conversely, the number of particles in the 10 to 20 µm range showed a significant reduction. This is explained by the continued disintegration between 5 and 15 minutes, and possibly by the dissolution of the material.

## Discussion

Previous studies have emphasized the importance of the specific surface area of particles or the tablet matrix after its disintegration in the human gastrointestinal tract, highlighting a critical parameter that influences drug solubility and absorption. The contact area between the drug and the solvent is directly proportional to the square of the granule surface, which has a direct impact on the drug's dissolution rate [8]. The results of this study demonstrate statistically significant differences in particle size for oral tablets containing diosmin-/hesperidin, which include purified and micronized flavonoid fractions (1000mg) commercially available in the Brazilian market. These findings suggest disparities in the quality of the micronization process among the evaluated medications, consistent with Zupanets et al. [7], who evaluated formulations commercially available in Europe, more specifically in France and Ukraine.

One significant finding is that particle size distribution also has a substantial influence on solubility, consequently affecting the rate and extent of drug absorption. It is plausible that reducing particle size increases this ratio, thereby enhancing the drug's solubility and dissolution rate and potentially leading to a faster, more complete attainment of the maximum plasma concentration [15]. This finding has significant implications for future research on the impact of MPFF micronization on key pharmacokinetic and pharmacodynamic parameters; however, the present study identified an important finding that distinguishes diosmin/hesperidin medications.

This investigation provided a detailed analysis of the particle size distribution of micronized diosmin/hesperidin tablets commercially available in the Brazilian market. The results obtained showed significant differences in granulometry among the analyzed medications, suggesting variations in the quality of the micronization process employed in the production of these formulations. Based on the findings, it can be stated that product I presents the most favorable particle size distribution for rapid tablet disintegration and dissolution, outperforming other medications.

The particle size distribution plays a crucial role in drug solubility and absorption, directly impacting therapeutic efficacy. Reducing particle size can result in a larger surface area available for interaction with the solvent, promoting faster and more complete drug dissolution, and consequently more efficient absorption in the body. However, differences can arise in the production of micronized particles, which, depending on the production method, may result in agglomerates. The particle analysis results were presented in the form of tables and graphs, highlighting particle statistics and size distribution. Particle analysis using ImageJ software proved to be an effective, robust, and easily accessible tool for routinely quantifying particles in pharmaceutical analysis. However, it is important to note that the accuracy of the analysis can be influenced by various factors, including image quality and the segmentation parameters used. Based on this, it is stated that any attempts to reproduce the results demonstrated here should consider the high rigor applied in all methodological stages as described in this work.

To compare the degree of micronization of flavonoid fractions from different manufacturers, the authors first used the laser diffraction technique, which revealed various methodological limitations. A primary disadvantage of this experimental method lies in the possible introduction of bias by the evaluation algorithm adopted, which is based on Fraunhofer diffraction theory for spherical particles, as indicated by Mühlenweg and Hirleman [16]. Such an approach can lead to inaccuracies in measuring irregularly shaped particles due to the influence of the particle's microstructure on the obtained results. Therefore, in the context of comparing micronization levels in flavonoid fractions using laser diffraction, it is imperative to consider the potential biases introduced by the variability of excipients among formulations, measurement techniques, and particle shape. Additionally, variations in equipment calibration, settings, and data interpretation can create inconsistencies, making direct comparisons between analyzed manufacturers challenging. For these reasons, the laser diffraction methodology was excluded from the study.

The findings of this study highlight the importance of precise granulometric characterization in evaluating the quality and consistency of micronized pharmaceutical formulations. Additionally, they underscore the need for stricter and standardized regulations on micronization processes in the pharmaceutical industry to ensure uniformity and efficacy of products available to patients.

## Conclusions

The present research aimed to evaluate the granulometric distribution of micronized purified flavonoid fraction (MPFF) tablets containing diosmin/hesperidin 1000 mg, commercially available in the Brazilian market, from four different manufacturers, after their spontaneous disintegration. The results showed significant disparities in particle size distribution among the products, with the reference product (product I) from Servier Laboratories standing out for having a higher proportion of particles in the 1 to 5 µm range. This more favorable granulometry suggests faster and more efficient absorption, reinforcing the importance of proper micronization to improve drug solubility and bioavailability. The methods employed, including optical microscopy and image analysis using ImageJ software, proved effective in the granulometric characterization of the products. The statistical analyses corroborated the findings, indicating significant differences between the products, especially in particles smaller than 5 µm, which are considered crucial for optimizing oral drug absorption.

This study contributes to understanding the relationship between the manufacturing process and the therapeutic performance of MPFF formulations (1000 mg). The observed variations highlight the need for standardization of micronization processes to ensure consistent quality and efficacy of the products available on the market. The adoption of stricter regulations and the encouragement of granulometric characterization technologies are fundamental steps to ensure the quality of medicines and, consequently, therapeutic efficacy, benefiting both the scientific community and patients.
